# Impact of the pandemic on hospital care for chronic pain patients in Germany

**DOI:** 10.3389/fmed.2024.1393855

**Published:** 2024-09-11

**Authors:** Thomas Cegla, Sven Hohenstein, Andreas Bollmann, Vincent Pellissier, Veronika Bencheva, Sven Schmiedl

**Affiliations:** ^1^Department of Pain Medicine, Helios University Hospital Wuppertal, Wiuppertal, Germany; ^2^Chair of Anasthaesia, School of Medicine, Faculty of Health, University of Witten/Herdecke, Witten, Germany; ^3^Leipzig Heart Center, Leipzig, Germany; ^4^Real World Evidence and Health Technology Assessment at Helios Health Institute, Berlin, Germany; ^5^Center for Clinical Trials, Faculty of Health, Witten/Herdecke University, Witten, Germany; ^6^Department of Clinical Pharmacology, School of Medicine, Faculty of Health, Witten/Herdecke University, Witten, Germany; ^7^Philipp Klee-Institute for Clinical Pharmacology, Helios University Hospital Wuppertal, Wuppertal, Germany

**Keywords:** COVID-19 pandemic, pain, inpatient treatment, chronic pain, undertreated pain, undertreatment of pain, undertreatment, COVID-19

## Abstract

**Objectives:**

The COVID-19 pandemic affected patients’ access to health services, including patients with severe chronic pain. Since limited data on pandemic-caused changes in pain therapy is available, we analyzed its effect on hospital-based pain treatment.

**Methods:**

For this retrospective claims data analysis conducted in *n* = 37 hospitals, we included patients treated for a chronic pain-related diagnosis. Discharge rates stratified by region and pain unit size were analyzed for different time periods between January 2019 and June 2022.

**Results:**

There was a significant decrease in day-care, inpatient interdisciplinary multimodal pain management, from a total of 5,533 hospital pre-pandemic treatments in 2019, to 3,942 in 2020 and 4,262 in 2021, with a slight increase in the first half of 2022. The extent of COVID-19-related changes differed depending on region and pain unit size.

**Conclusion:**

The decreased number of hospital pain treatments during the pandemic implies a relevant analgesic undertreatment. During future pandemics, the ethical dimension of potentially non-sufficient pain treatment should be weighted against social, medical and hygienic restrictions influencing the hospitalization rate.

## Introduction

The COVID-19 pandemic led to worldwide restrictions on citizens’ freedom and changed access to health services, affecting not only acute medicine but also the care of chronically ill individuals, including those suffering from chronic pain, which affects patients worldwide (1). Chronic non-tumor related pain has a high prevalence in several countries, including Germany (2–9). The influence of the COVID-19 pandemic on the health care of patients with documented chronic pain during the pandemic has been described in several countries patients around the world (1). An impact on the different determinants of health could affect pain patients more (6, 10). The COVID-19 pandemic had an impact on mental health of people with pre-existing psychiatric disorders, but also led to mental distress in others (11–14). The social distance had an impact on mental stability, well-being, and health, and thus had medical consequences (15). COVID-19 associated challenges arose in several medical care settings, were sometimes unexpected but had also an impact on pain treatment. For example, the availability of several drug classes like antidepressants or opiates was limited by supply shortages (16). In addition, different regional and federal state policies during different pandemic phases had also an impact on the care for pain patients (17, 18).

Since its introduction into medicine, the bio-psycho-social model of a disease (19, 20) has become important and established in clinical practice (21). For patients with chronic pain the bio-psycho-social model of illness is used as the basis for care, and it has an impact on the design of inpatient interdisciplinary multimodal pain therapy (IMMST). A multimodal pain therapy that includes psychological and social factors is more effective than a purely medical treatment approach, but requires accompanying critical consideration with regard to individuality (22, 23). Interdisciplinary interprofessional pain conferences are an important indispensable component of multimodal pain management in hospitals (24, 25). For several patient groups, e.g., patients with back pain, benefits were clearly shown (24). Patients eligible for IMMST are those with chronic pain whose quality of life and/or ability to work is limited despite outpatient pain therapies, and who have additional illnesses that need to be taken into account for treatment or who have misused analgesic drugs. IMMST plays an important role in the care. Of interest are the potential changes in inpatient pain management care during the pandemic progresses. Pandemic-related treatment interruptions in pain management with effects on pain control, quality of life, and course have been reported (26). All parts of the bio-psycho-social model of chronic pain have been affected.

Chronic pain is a chronic condition and requires both outpatient care and the possibility of inpatient treatment. Because the pandemic changed key factors in outpatient care, and because pain patients, often with co-occurring mental health conditions, were more severely affected by the overall situation, it is likely that more inpatient care was needed during this period. However, this presumed need coincides with an overall situation of reduced in-patient treatment capacity. Although there was a greater need for in-patient treatment, the treatment options were severely limited in terms of treating patients with severe pain sufficiently. In addition, there was a general feeling of insecurity among the population at various stages, which was also associated with a reluctance to visit health care facilities. To summarize the overall impact of these conflicting trends and taking into account scare data for COVID-19 associated changes in treatment of patients with severe chronic pain, we aim to analyze the effect of COVID-19 pandemic on hospital-based pain treatment in our study.

## Methods

### Data source

Claims data from *n* = 37 hospitals of the largest private German hospital group (i.e., Helios) fulfilling the inclusion criteria defined below were used for this retrospective analysis.

### Definition of patients

#### Inclusion criteria

Patients were included, if they were discharged from hospital between 2019-01-01 and 2022-06-30 following an in-patient or day care patient (admission due to one of the following diagnosis-related group (DRG) codes):

- B47A: Multimodal pain therapy for diseases and disorders of the nervous system at least 14 treatment days
- B47B: Multimodal pain therapy for diseases and disorders of the nervous system less than 14 treatment days
- I42A: Multimodal pain therapy for diseases and disorders of musculoskeletal system and connective tissues at least 14 treatment days
- I42B: Multimodal pain therapy for musculoskeletal and connective tissue diseases and disorders less than 14 treatment days
- U42A: Multimodal pain therapy for mental diseases and disorders, age under 19 years
- U42B: Multimodal pain therapy for mental diseases and disorders at least 14 treatment days, age over 18 years
- U42C: Multimodal pain therapy for mental diseases and disorders less than 14 treatment days, age over 18 years.

Since these codes take into account not only the chronic pain but also the main cause, there are three codes, one each for the musculoskeletal system, the nervous system, and psychological and somatoform disorders. Minimum lengths of hospital stay of at least 14 treatment days or under 14 treatment days are coded separately, and the codes are doubled to 6, allowing the number of IMMST treatments over time to be shown using the DRG coding. By comparing the number of different IMMST DRGs before and during the pandemic, changes in care can be determined. To obtain a more precise representation, the Helios data set was analyzed, revealing changes in care in different federal states but also showing that care was provided at all times in the Helios hospitals, albeit at a reduced level, especially during lockdowns, at the beginning of the pandemic, and under the incentive of freehold flat rates.

#### Exclusion criteria

Cases with several DRG codes (4 cases in total) due to case merging by hospital controlling were removed to avoid double counting patients/cases. For this study, no further exclusion criteria (e.g., patients’ age) were applied.

### Comparisons

Patient characteristics considered in our analysis were age, age group, gender, Elixhauser weighted score (AHRQ algorithm was applied (27, 28)) and corresponding comorbidities. Uncomplicated and complicated diabetes were lumped into one single category, as were uncomplicated and complicated hypertension.

Comparisons of discharged patients, discharged cases (hospital stays), and discharge rates were made for different time periods according to the following definitions:

Annual comparison: Discharge rates were analyzed for the years 2019, 2020, 2021, and 2022. For 2022, only the first six months (January to June) were included due to limited data availability.

Quarterly comparisons: Quarterly discharge rates in 2020, 2021 and 2022 were compared to the respective quarter in 2019 to minimize annual effects. As the first quarter of 2020 overlap both periods (prepandemic and pandemic), results for this comparison (Q1 2020 vs. Q1 2019) should be treated with caution.

Prepandemic vs. pandemic: Prepandemic period is the time interval between 2019-01-01 and the week including 2020-02-28 whereas the pandemic period is the time interval between the week starting after 2020-02-28 and 2022-06-30. We have defined the beginning of the pandemic period according to the date when the first patients with COVID-19 were detected in two large German federal states (i.e., Baden-Wuerttemberg, North Rhine-Westphalia) on the 26^th^ of February 2020.^1^ The end date of the pandemic period was defined taking into account the distinct decrease of COVID-19 cases and associated healthcare utilization in the second quarter of 2022 (29).

### Stratification

Analyses were startified for regions (federal states of Germany) and pain units size. Pain unit size were defined as follows, (i) ‘high volume’: units with at least 300 cases in 2019 and (ii) ‘low volume’ with less than 300 cases according to the aforementioned DRG definitions.

### Assessment of comorbidities

For assessing the comorbidities of patients, the AHRQ Elixhauser Comorbidity Index was used (28). For this index, comorbidites were defined via patients’ ICD codes and entered the index with different weights. The index was evaluated with respect to in-hospital mortality and readmissions.

### Statistical analysis

For the analysis of patient characteristics, categorical variables were analyzed using χ2 test, and two-sample t-test was used for analyzing continuous variables.

For the comparison of the discharge rate between different cohorts, a Poisson GLMMs with log-link function and the hospitals and a random intercept (30) was used. Hospitals entered the analyses as random factor. We analyzed weekly numbers for linear trends over time with Poisson regression. Discharge rates are reported as mean weekly discharge (SD), incidence rate ratios (IRR) together with 95% confidence intervals and *p* values. For weekly data, IRRs indicate average weekly changes. Stratified analyses were conducted for DRG and hospital regions. For all tests we applied a two-tailed 5% error criterion for significance. All the analyses are patient-based analyses (and not case-based). In case of multiple admissions for a patient (which appears to be rather rare), only the first case was included in our analysis.

Inferential statistics were performed in the R environment for statistical computing (version 4.0.2), 64-bit build (31) Effects of mixed models were estimated with the lme4 package (version 1.1–26) (30, 32).

## Results

### Patient characteristics

Comparing the characteristics of patients discharged during the observed pandemic versus pre-pandemic period, only minor differences were found (Table 1). During the pandemic period, patients were slightly younger (mean age: 60.3 ± 13.8 yrs. vs. 61.0 ± 13.6 yrs., *p* < 0.01) and had a slightly higher mean Elixhauser score (−2.0 ± 5.8 vs. −2.2 ± 6.0, *p* < 0.05). With regard to comorbidities used for defining the Elixhauser score, a slightly increased proportion was found during the pandemic period for patients suffering from ‘other neurological disorders’ (3.4% vs. 2.8%, *p* < 0.05) whereas a decreased proportion of patients had a documented diagnosis of obesity (23% vs. 27%, *p* < 0.001), depression (46% vs. 48%, *p* < 0.05), and hypertension (50% vs. 52%, *p* < 0.01).

**Table 1:** Patient characteristics (pre-pandemic vs. pandemic).

	Pre-pandemic	Pandemic	*p* value
*N*	6,248	7,889	
Age			
Mean (SD^1^)	61.0 (13.6)	60.3 (13.8)	<0.01
≤ 59 years	3,113 (50%)	4,011 (51%)	<0.01
60–69 years	1,334 (21%)	1,773 (22%)	
70–79 years	1,162 (19%)	1,296 (16%)	
≥ 80 years	639 (10%)	809 (10%)	
Sex			
Female	4,379 (70%)	5,495 (70%)	0.578
Elixhauser score			
Mean (SD^1^)	-2.2 (6.0)	−2.0 (5.8)	<0.05
< 0	4,035 (65%)	4,835 (61%)	<0.001
0	934 (15%)	1,417 (18%)	
1–4	535 (8.6%)	693 (8.8%)	
≥ 5	744 (12%)	944 (12%)	
Elixhauser comorbidities			
Congestive heart failure	308 (4.9%)	420 (5.3%)	0.292
Cardiac arrhythmias	544 (8.7%)	637 (8.1%)	0.177
Valvular disease	179 (2.9%)	188 (2.4%)	0.074
Pulmonary circulation disorders	28 (0.4%)	38 (0.5%)	0.771
Peripheral vascular disorders	308 (4.9%)	341 (4.3%)	0.087
Paralysis	126 (2.0%)	148 (1.9%)	0.547
Other neurological disorders	176 (2.8%)	269 (3.4%)	<0.05
Chronic pulmonary disease	812 (13%)	993 (13%)	0.469
Hypothyroidism	981 (16%)	1,174 (15%)	0.178
Renal failure	818 (13%)	986 (12%)	0.293
Liver disease	186 (3.0%)	200 (2.5%)	0.109
Peptic ulcer disease excluding bleeding	9 (0.1%)	4 (<0.1%)	0.069
AIDS/HIV^2^	0 (0%)	0 (0%)	
Lymphoma	9 (0.1%)	24 (0.3%)	0.050
Metastatic cancer	9 (0.1%)	7 (<0.1%)	0.331
Solid tumor without metastasis	62 (1.0%)	74 (0.9%)	0.743
Rheumatoid artritis/collaged vascular disease	471 (7.5%)	593 (7.5%)	0.961
Coagulopathy	81 (1.3%)	77 (1.0%)	0.072
Obesity	1,684 (27%)	1,819 (23%)	<0.001
Weight loss	77 (1.2%)	83 (1.1%)	0.314
Fluid and electrolyte disorders	111 (1.8%)	139 (1.8%)	0.948
Blood loss anemia	4 (<0.1%)	4 (<0.1%)	0.738
Deficiency anemia	60 (1.0%)	83 (1.1%)	0.588
Alcohol abuse	128 (2.0%)	144 (1.8%)	0.337
Drug abuse	337 (5.4%)	482 (6.1%)	0.070
Psychoses	22 (0.4%)	30 (0.4%)	0.784
Depression	2,982 (48%)	3,631 (46%)	<0.05
Diabetes	1,065 (17%)	1,252 (16%)	0.061
Hypertension	3,253 (52%)	3,909 (50%)	<0.01

(1) SD, Standard Deviation rivation; (“) AIDS/HIV, Acquired Immunodeficiency Syndrome/Human Immunodeficiency Virus.

Starting from a total of 5,533 cases in 2019, the COVID-19 pandemic reduced the number of cases to 3,942 in 2020 and to 4,262 in 2021. Hence, in 2020 the most significant decrease was recorded in day-care, inpatient interdisciplinary multimodal pain therapy. In 2021, there was a slight increase which, however, did not reach the level of care in 2019. The same is true for the first half of 2022 (Table 2).

**Table 2:** Number of cases and patients, stratified by DRG type and discharge year (2019, 2020, 2021, 01–06/2022).

DRG^1^	Cases					Patients				
	Total	2019	2020	2021	2022*	Total	2019	2020	2021	2022^2^
*N*	15,907	5,533	3,942	4,262	2,170	14,137	5,386	3,883	4,187	2,159
B47										
B47Z	1,796	597	473	505	221	1,637	586	467	499	220
B47A	1,335	402	375	387	171	1,212	392	372	384	171
B47B	461	195	98	118	50	448	194	98	117	49
I42										
I42Z	8,514	2,829	2,146	2,314	1,225	7,827	2,785	2,120	2,271	1,219
I42A	5,189	1,533	1,244	1,593	819	4,766	1,517	1,237	1,568	818
I42B	3,325	1,296	902	721	406	3,169	1,276	896	715	405
U42										
U42A	2	1	0	1	0	2	1	0	1	0
U42B	4,506	1,768	1,029	1,117	592	4,154	1,746	1,020	1,107	592
U42C	1,089	338	294	325	132	1,065	338	293	324	131

(1) DRG, diagnosis-related group; DRG codes ending in-Z are a combination of DRG code with the same first 3 letters/number. (2) 2022, only the first six months (January to June) were included due to limited data availability.

Regarding the whole study period, we found a trend for a decreased number of discharged patients for almost all DRGs (Table 3). By separating the two main period (pre-pandemic vs. pandemic) we found for the majority of DRGs a significant increase of patients during the pandemic period underlining the sharp decrease at the beginning of the pandemic followed by a slight increase of treated patients.

**Table 3:** Weekly trends of patient numbers for the whole study period stratified by DRG type.

DRG^1^	Whole period		Pre-pandemic		Pandemic	
	IRR^2^ (95%CI^3^)	*p*-value	IRR^2^ (95%CI^3^)	*p*-value	IRR^2^ (95%CI^3^)	*p*-value
(Total)	0.997 (0.997; 0.997)	<0.001	0.998 (0.997; 1.000)	<0.05	1.003 (1.003; 1.004)	<0.001
B47Z	0.997 (0.996; 0.998)	<0.001	1.000 (0.995; 1.004)	0.918	1.003 (1.002; 1.005)	<0.001
B47A	0.999 (0.997; 1.000)	<0.05	1.005 (1.000; 1.011)	0.070	1.004 (1.001; 1.006)	<0.01
B47B	0.994 (0.992; 0.996)	<0.001	0.989 (0.981; 0.997)	<0.01	1.003 (0.999; 1.007)	0.125
I42Z	0.998 (0.997; 0.998)	<0.001	0.998 (0.996; 1.000)	0.092	1.003 (1.002; 1.004)	<0.001
I42A	1.000 (0.999; 1.000)	0.158	1.003 (1.000; 1.006)	<0.05	1.005 (1.004; 1.006)	<0.001
I42B	0.994 (0.994; 0.995)	<0.001	0.993 (0.990; 0.996)	<0.001	1.000 (0.998; 1.001)	0.688
U42A	0.995 (0.968; 1.023)	0.741	0.972 (0.859; 1.099)	0.646	1.012 (0.953; 1.075)	0.689
U42B	0.995 (0.995; 0.996)	<0.001	0.998 (0.995; 1.000)	0.082	1.004 (1.003; 1.005)	<0.001
U42C	0.998 (0.997; 0.999)	<0.01	0.999 (0.993; 1.005)	0.785	0.999 (0.997; 1.001)	0.331

(1) DRG, diagnosis-related group; DRG codes ending in-Z are a combination of DRG code with the same first 3 letters/number. (2) IRR, Incidence Rate Ratio; (3) Cl, confidence interval.

### Regional differences (federal states)

In our analysis, we found some regional differences at level of the federal states of Germany. Saxony-Anhalt has shown the smallest changes of discharged pain patients over the course of the pandemic (Table 4).

**Table 4:** Number of cases stratified by federal states of Germany and discharge year.

Federal state	2019	2020	2021	2022
Bavaria	392	230	251	176
Berlin	142	58	49	41
Brandenburg	191	76	94	82
Hesse			14	
Lower Saxony	1,164	802	912	411
North Rhine-Westphalia	2,173	1,268	1,459	739
Saxony	584	381	254	132
Saxony-Anhalt	720	640	702	337
Schleswig Holstein	96	51	27	3
Thuringia	71	436	500	249

### Pain unit size

With regard to the total discharge rates, we found statistically significant differences between the pre-pandemic and pandemic period for both, low and high volume pain units (Table 5). In a combined analysis of all selected DRGs, a more pronounced decrease was revealed for high volume pain units [incidence rate ratio [IRR]: 0.63 (95% CI: 0.59 to 0.66)] compared to low volume units [IRR: 0.90 (95% CI: 0.86 to 0.94)] between the two periods for the DRGs (Table 5).

**Table 5:** Comparison of patient discharge rates, stratified by DRG and pain unit size.

	Weekly patients (mean [SD])								
	Low volume units				High volume units				Interaction (low vs. high volume units, pandemic vs. pre-pandemic)
DRG^1^	Pre-pandemic	Pandemic	IRR^2^ (95%CI^3^)	*p*	Pre-pandemic	Pandemic	IRR^2^ (95%CI^3^)	*p*	*p*
(Total)	4.12 (2.8)	3.71 (2.71)	0.90 (0.86; 0.94)	<0.001	10.65 (5.45)	6.63 (4.04)	0.63 (0.59; 0.66)	<0.001	<0.001
B47Z	0.44 (0.79)	0.38 (0.78)	0.87 (0.76; 0.99)	<0.05	1.11 (1.8)	0.84 (1.59)	0.78 (0.66; 0.92)	<0.01	0.461
B47A	0.23 (0.61)	0.25 (0.68)	1.07 (0.89; 1.27)	0.471	1.05 (1.75)	0.75 (1.55)	0.74 (0.62; 0.87)	<0.001	<0.01
B47B	0.21 (0.55)	0.13 (0.41)	0.65 (0.52; 0.80)	<0.001	0.06 (0.27)	0.09 (0.4)	1.53 (0.82; 2.86)	0.181	<0.01
I42Z	2.42 (2.42)	2.26 (2.48)	0.85 (0.81; 0.90)	<0.001	4.34 (3.75)	3.05 (3.2)	0.69 (0.64; 0.76)	<0.001	<0.001
I42A	1.11 (2.17)	1.26 (2.38)	0.93 (0.86; 1.01)	0.093	3.32 (3.08)	2.5 (2.93)	0.75 (0.68; 0.83)	<0.001	<0.01
I42B	1.31 (1.81)	1 (1.51)	0.77 (0.71; 0.84)	<0.001	1.03 (1.87)	0.56 (1.17)	0.51 (0.43; 0.62)	<0.001	<0.01
U42A	0 (0)	0 (0.03)		0.830	0 (0.07)	0 (0)		0.999	
U42B	0.92 (1.69)	0.72 (1.33)	0.92 (0.83; 1.01)	0.080	4.94 (5.05)	2.45 (2.89)	0.50 (0.46; 0.55)	<0.001	<0.001
U42C	0.33 (0.93)	0.35 (1.02)	1.34 (1.14; 1.57)	<0.001	0.25 (0.52)	0.29 (1.1)	1.16 (0.84; 1.58)	0.367	0.685

The data of four clinics had to be removed because of missing 2019 data. (1) DRG, diagnosis-related group; DRG codes ending in-Z are a combination of DRG code with the same first 3 letters/number. (2) IRR, incidence rate ratio, (3) CI, confidence interval.

With regard to single DRGs, a more complex picture was found. There are three codes, one each for the musculoskeletal system, the nervous system, and psychological and somatoform disorders. Minimum lengths of hospital stay of at least 14 treatment days or under 14 treatment days are coded separately. These 6 codes allowing the number of IMMST treatments over time to be shown using the DRG coding. Whereas for most DRGs a decreased discharge rate was found for both, high and low volume units comparing the pre-pandemic and the pandemic period, discrepant results were found for B47A and B47B. For both unit types, an increased IRR (i.e., increased discharge rate comparing pre-pandemic and pandemic period) was found for U42 C.

## Discussion

In our study, we found that there was a substantial decrease in the number of patients hospitalized for severe pain during the COVID-19 pandemic.

### Changes in hospital treatment

Changes in hospital care for patients with chronic pain occurred as a result of the COVID-19 pandemic. There was a significant decrease in inpatient multidisciplinary multimodal pain care, from a total of 5,533 hospitalizations in 2019 before the pandemic to 3,942 in 2020 and to 4,262 in 2021. There was a slight increase in the first half of 2022. Overall, however, these figures are also lower than the pre-pandemic figures from 2019.

### Patient populations and regional variation

In our study, we found some small but statistically significant differences between the two cohorts. For example, patients of pandemic cohort were slightly younger and had a slightly higher Elixhauser Score than pre-pandemic patients. These results suggest a shift of treating severe pain under in-hospital conditions from elderly patients to younger, sicker patients (i.e., suffering from a higher number of comorbidities). However, these difference are small and of questionable clinical relevance.

Regarding regional variation, it’s worth mentioning that there are 16 federal states in Germany responsible for the regional hospital care. The measures to contain the pandemic were not the same in all of them. However, Table 4 provides an overview of the absolute care figures by federal state. Discussing all different regional COVID-19 related measures and their impact on our study results would be far beyond of our study topic.

### The bio-psycho-social disease model under tightened hygiene measures

The multimodal inpatient and day-care treatment of chronic pain patients is based on the bio-psycho-social disease model and is an important component of pain medicine care. However, because of patient-centered therapies and participation in treatment groups, IMMST was very limited during the different phases of the pandemic due to stricter hygiene measures. This was compounded by uncertainty and anxiety among patients and their families, which led to a decline in demand for this healthcare service.

### General conditions

Emergency treatments and treatments that could not be postponed in the hospital were prioritized (33). In addition to these medically sound care decisions, there were also economic incentives in Germany to keep bed capacities free for patients infected with COVID-19 or to use the remaining capacities as economically as possible (18). Staff reassignments were also necessary to care for wards with a focus on COVID-19 patients, intermediate care, and intensive care units.

### The impact of the COVID-19 pandemic on the pain patient

The experience of psychosocial and spiritual aspects of palliative care can be used to evaluate pain care under COVID-19 conditions (34, 35). The pandemic had an impact on the components of the bio-psycho-social disease model of chronic pain patients. However, mood changes, social isolation, physical inactivity, and changes in dietary habits affected all populations to varying degrees. When pain patients have less contact with medical providers, it affects quality of life and pain severity. The risk of pain becoming chronic may be greater under the impact of a pandemic (35, 36). The pandemic has been and continues to be a psychosocial challenge for society and individuals. Coping strategies were different and should be the basis for reorienting medical action. The basis of pain management is the holistic approach, which is also required in psychosomatic treatment (1, 4, 24, 37). The pandemic has had a significant impact on patients with chronic pain. With increasing instability and chronification of pain and insufficient outpatient treatment options, an increase in multimodal inpatient treatment would have been logical. However, this did not happen because of the restrictive hygiene regulations and the focus of clinical treatment on COVID-19 patients.

### Pain unit size and interdisciplinary multimodal pain management (IMMST) under tightened hygiene measures

Despite all the aforementioned legal restrictions and other challenges related to COVID-19, pain medicine care has taken place in hospitals. Even under these aggravated conditions, multimodal pain management has been implemented (38).

In our anlysis, a more pronounced decrease of patients receiving multimodal pain treatment was revealed for high volume pain units compared to low volume units. This might be related to the fact, that larger pain clinics or wards were more difficult to organize IMMST because these hospitals required more staff to care for critically ill COVID-19 infected patients and had to deal with staff absences due to sick leaves.

Whereas for most multimodal pain therapy DRGs, a decrease of discharge rates were found comparing pre-pandemic and pandemic period, an increase was found for selected DRGs (B74A: low volume units, B74B: high volume units). In other words, at least for ‘Multimodal pain therapy for diseases and disorders of the nervous system’ we found an increase of ‘short term treatments’ (less than 14 days) in low volume units whereas in high volume units, an increase of ‘long term treatment’ (at least 14 days) was found. Hence, we hypothesize a “centralisation” of severe (and long term cases) in high volume units.

Interestingly, discharge rates for multimodal pain therapy for mental diseases and disorders were increased for both unit types underlining the treatment need of pain and psychiatric comorbidities during the COVID-19 pandemia.

In addition to the problems of inpatient care, options in outpatient care for pain patients were also limited (39). Patients visited primary care physicians and specialists less frequently. This resulted in a lack of the usual hospital admissions. Desired treatments were postponed, and there was a general reluctance on the part of patients to undergo inpatient therapies. Skepticism and fear of infection when in contact with medical facilities were major issues here. An expected larger number of patients suffering from chronic pain due to inadequate care, as well as pandemic-related health restrictions in the biological, psychological, and social spheres due to lock-related movement restrictions, generally depressed mood, and reduced social contacts, should actually have led to a significant increase in inpatient IMMST after the end of the pandemic care restrictions, according to our expectations. However, this was not the case in the clinics studied. Overall, IMMST occurred despite the pandemic. The continued decline could be a temporary phenomenon. Another interpretation is that the perception of this form of treatment by referring physician colleagues has decreased. The clinics studied care for patients in different ways, some caring for more patients than before the pandemic, but others caring for fewer. The high demands placed on the structure of an IMMST can be better met by larger units of care (7, 40, 41).

### Has the COVID-19 pandemic led to more chronic pain patients?

Data from orthopedic practices show an increase in new neck and back pain diagnosed during the pandemic (42). There are some data showing that delay or even failure to diagnose pain-related conditions may lead to an increased risk of developing chronic pain (42). Periods of crisis that affect the psychological stability of people with chronic pain lead to a higher risk of exacerbation of the disease (32). Our study does not provide evidence that a greater number of patients are hospitalized for multimodal pain management after the pandemic. However, because patients with complex pain syndromes often receive frustrating therapy for an extended period of time, a general pandemic-related increase in complex pain treatments, which may occur somewhat later, cannot be ruled out at this time. It is possible, however, that a shift of complex pain treatments to the outpatient setting could also lead to a situation in which any increased post-pandemic need for care is not reflected in the inpatient setting.

### Pain management under difficult conditions

We were able to show that pain medicine care in hospitals took place and was possible during the COVID-19 pandemic. This is important because a combination of methods is also necessary for the individual components of multimodal pain therapy, as the example of psychotherapy shows (43). The DRG-based reimbursement system is also viewed critically in terms of utilization and efficiency, even in international comparison (44, 45). This and the differences in care limit international comparability. In addition to inpatient treatment of severe pain, outpatient interdisciplinary pain treatment is also possible under certain conditions (2). Promising results have been shown, for example, for patients with chronic nonspecific back pain (10). We did not investigate medical care in this area.

### COVID-19 and pain: future research needs

Even after recovery from an acute COVID-19 infection, several pain-related long-term complaints were reported in post-COVID and long-COVID patients. For example, testicular pain, headache, chronic pain, and chest pain were reported as long-term pain syndromes (46). Hence, preventive measures are necessary to prevent chronic concomitant diseases, such as the chronic pain of COVID-19 infection (47). Vaccinations leading to an activation of antiviral immunity are preventive measures, as are presumably also the improvement of vitamin D deficiency and the intake of other micronutrients (48). However, further research is needed to fully understand and optimize treatment new onset and long lasting pain in COVID-19 patients as well as deterioration of chronic pain syndromes in patients with a COVID-19 infection. Furthermore, there is also a huge need for research in view of the emergence of new COVID variants or future pandemics which may build on the knowledge gained during the first pandemic waves.

### Strengths

A particular strength of our study is the large number of included hospitals, which includes a variety of different departments in different regions of Germany and reflects the diversity of the German hospital landscape. Especially the avoidance of a one-sided focus on specialized pain centers and the large number of included patients underlines the high external validity of our results.

### Limitations

As with any observational study using electronic hospital data, undercoding and miscoding of diagnoses must be expected. In addition, changes in the population during the observation period (hospitals newly admitted to the Helios Group or department closures) may also influence the absolute number of patients and cases considered. However, the case numbers of Helios hospitals under consideration can also be influenced by decisions of other hospital operators, such as possible new openings/closures or changes in the department structure. Since not all hospitals in Germany are included in the data, this limitation exists.

Against this background, the use of incidence rates in our study is explained in order to minimize such fluctuations in the population as far as possible.

Due to the data structure and because of data protection regulations, linking patient data between different hospitals was not possible. This limitation implies the possibility of double-counting specific patients who received treatment at multiple hospitals.

## Conclusion

What can we learn for the future care of pain patients? There is an ethical dimension to the treatment of pain and how society deals with it (11, 39, 49). To date, this has not been considered in the care of patients. This is also reflected in the possible inadequate consideration and undertreatment of pain during the COVID-19 pandemic and a possible next one. Implications for future research include continued monitoring of inpatient multimodal treatment for pain and outpatient therapy after the official end of the pandemic. Whether the number of pain patients will increase as a result of the COVID-19 pandemic is also worth investigating.

## Data Availability

The data analyzed in this study is subject to the following licenses/restrictions: Raw data can not be provided due to data protection laws. Requests to access the aggregated datasets should be directed to Sven Hohenstein, sven.hohenstein@helios-health-institute.com.

## Footnotes

^1^ https://www.bundesgesundheitsministerium.de/coronavirus/chronik-coronavirus
